# Male sex is an independent risk factor for patellar osteochondral fractures following acute patellar dislocation in pediatric patients

**DOI:** 10.1186/s40634-023-00646-4

**Published:** 2023-08-26

**Authors:** Julia S. Retzky, Tyler J. Uppstrom, Danielle E. Chipman, Patawut Bovonratwet, Daniel W. Green

**Affiliations:** Department of Pediatric Orthopaedic Surgery, Hospital for Special Surgery, 535 E 70th Street, New York, NY 10021 USA

## Abstract

**Purpose:**

Although most patellar dislocations are associated with medial patellofemoral ligament (MPFL) injury, many patients also sustain concomitant patellar osteochondral fractures following a patellar dislocation. Few prior studies have described or evaluated risk factors for patellar osteochondral fractures in pediatric patients. The purpose of the present study was to describe the incidenceand location of patellar osteochondral fractures following acute patellar dislocation in pediatric patients. In addition, we described risk factors for patellar osteochondral fractures in this population. We hypothesized that most fractures would occur at the inferomedial quadrant of the patella following a traumatic injury mechanism.

**Methods:**

Following Institutional Review Board approval, the electronic medical record was queried to identify pediatric patients ≤ 18 years old who underwent MPFL reconstruction (MPFLR) or non-operative treatment for patellar instability between July 2016 and February 2020. Osteochondral fractures were defined as full-thickness chondral injuries with attached subchondral bone or purely osseous injuries measuring ≥ 3 mm (mm) in at least two magnetic resonance imaging (MRI) planes. Patients were included in the study if they had complete preoperative x-ray and MRI studies with minimum 6-month follow-up. Patients were excluded if they had incomplete imaging, isolated chondral fractures, or less than 6 months follow-up. Univariate analysis was used to identify patient factors associated with osteochondral fractures. Multivariate regression analysis was used to identify risk factors for osteochondral fractures.

**Results:**

Sixty patients (63 knees) were included in the study, 15 (23.8%) of whom had a patellar osteochondral fracture. The majority of osteochondral fractures (87%) involved the inferomedial quadrant of the patella. Univariate analysis showed an association between male sex (*p* = 0.041), skeletal immaturity (*p* = 0.028), and decreased patellar tilt (*p* = 0.021) and patellar osteochondral fractures. Multivariate regression analysis identified male sex as an independent risk factor for osteochondral fractures (relative risk: 4.8, 95% confidence interval [CI]: 1.08–20.9, *p* = 0.039). No patients had recurrent dislocation at minimum 6-month follow-up. All patients with osteochondral fractures returned to sports at most recent follow up.

**Conclusion:**

In this study, 23% of pediatric patients with acute patellar dislocations have a concomitant patellar osteochondral fracture. The majority of patellar osteochondral fractures involve the inferomedial quadrant of the patella. Male sex is an independent risk factor for patellar osteochondral fractures, and skeletal immaturity is associated with patellar osteochondral fractures in this population.

**Level of evidence:**

Level III.

## Introduction

Acute patellar dislocations (APDs) are a common cause of knee pain in pediatric patients [3]. Twenty-nine per 100,000 children will sustain at least one patellar dislocation between the ages of 10 and 17 [6], with 35% of these patients experiencing recurrent patellar dislocations [8]. Well-established risk factors for APD include trochlear dysplasia, increased tibial tubercle-to-trochlear groove (TT-TG) distance, increased lateral patellofemoral tilt, torsional malalignment of the femur/tibia, and patella alta [19].

The majority (96–97%) of APDs are associated with injury to the medial patellofemoral ligament (MPFL [8, 24]), which functions as the primary soft tissue restraint to lateral patellar translation at 0–30 degrees of knee flexion [11]. Although MPFL injuries are a common sequela of APDs, concomitant pathologies, including chondral or osteochondral injuries of the patella, trochlea, or lateral femoral condyle, and medial collateral ligament injuries, are also frequently associated with these injuries [4, 32].

The incidence and characteristic patterns of concomitant patellar osteochondral fractures in adults with APDs are well-described. Eleven to 76% of adults sustain osteochondral fractures following patellar dislocation, the majority of which (42 – 63%) involve the patella [15, 29, 30]. Mochizuki et al. [12] found that 84% of patellar osteochondral fractures in adults with APD were at the inferomedial border of the patella, whereas the minority of patients (16%) sustained superomedial patellar osteochondral fractures. Other studies [4, 25, 27] have similarly found that the majority of patellar osteochondral fractures involve the medial margin or inferomedial facet of the patella in mixed cohorts of pediatric and adult patients.

Patellar osteochondral fractures are a relatively common sequela of APD in children, with a reported incidence of 29% in first-time dislocation events [14]. A previous investigation demonstrated that the majority of chondral and osteochondral fractures associated with APD in pediatric patients involved the patella (versus the lateral femoral condyle), with the medial and inferomedial portions of the patella most commonly affected [10]. In addition, APDs associated with a traumatic mechanism were associated with a 42-time increased risk of chondral or osteochondral injury of the patella [10]. However, the aforementioned study did not separate grade IV chondral defects from true osteochondral fractures in their analyses. It is critical to evaluate outcomes following osteochondral injury in this patient population, as these injuries are often managed differently than pure chondral injuries. There is a paucity of information in the literature regarding the incidence and patterns of patellar osteochondral injuries following APD in pediatric patients. In addition, no prior studies have evaluated radiographic risk factors for patellar osteochondral fracture in pediatric patients with APD.

The primary aim of the present study was to describe the incidenceand location of patellar osteochondral fractures in pediatric patients with acute patellar dislocations. The secondary aim of the study was to describe risk factors for concomitant patellar osteochondral fracture, surgical management, and recurrent dislocation rate in this patient population at mid-term follow-up. The authors hypothesized that the majority of fractures would involve the inferomedial quadrant of the patella and occur more frequently following traumatic injury mechanisms.

## Materials and methods

### Patient selection

Institutional review board (IRB) approval was obtained at our institution prior to initiation of the study (IRB number: 2020–0203). We conducted a retrospective review of a consecutive series of pediatric patients treated for acute patellar dislocation at a single tertiary care center between July 2016 and February 2020. All surgical procedures were performed by a single pediatric orthopaedic sports medicine-trained orthopaedic surgeon (D.W.G.). Eligible patients were identified either by query of the electronic medical record by procedure type (MPFL reconstruction) and surgeon (D.W.G.) or ICD-10 code for patellar instability (M2200, M2201, M2202, M2210, M2211, M2212) from July 2016 to February 2020.

Patients were included in the study if they were ≤ 18 years old and underwent pre-operative anteroposterior, lateral, and Merchant view plain radiographs and standard magnetic resonance imaging (MRI) of the affected knee. Inclusion criteria also consisted of minimum 6-month postoperative follow-up for patients who underwent surgery or minimum 6-month clinical follow-up post-diagnosis if they elected to pursue non-operative treatment. Patients with incomplete imaging, isolated chondral fractures, or less than 6 months of postoperative/post-diagnosis clinical follow-up were excluded from analysis.

### Surgical technique for skeletally immature patients

Hamstring autograft was used for all patients. A 2 cm posteromedial incision is made over the proximal tibia, and a tendon harvester is used for semitendinosus tendon harvest. The graft is prepared for double-bundle reconstruction, with a target final graft length of 60 mm, including 20 mm in the femoral socket. A 3 cm anteromedial incision is made over the patella, and the medial border of the patella is exposed. Two patellar sockets are drilled in the proximal half of the patella to a depth of 12-15 mm. A 2 cm incision is made over the femoral MPFL insertion site. A guidewire is placed at Schöttle’s point [23] in the epiphysis of the distal femur aimed from medial to lateral, 5-7 mm distal to the physis.

A femoral socket is drilled to a depth of 20 mm. The doubled end of the graft is placed into the femoral socket and secured with an interference screw. The graft is then passed between layers 2 and 3 of the knee, and the graft length is determined based on the appropriate tension at 30 degrees of knee flexion. The two free ends of the graft are secured to the patellar sockets with suture anchors. The knee is brought through full range of motion for a final check of graft isometry [26].

### Surgical technique for skeletally mature patients

Like the skeletally immature patients, hamstring autograft was used for skeletally mature patients who had closed distal femoral physes on magnetic resonance imaging (MRI) using the same technique described above. The graft is prepared for double-bundle reconstruction, and the patellar sockets are prepared in the same fashion. A 2 cm incision is used at the MPFL insertion site on the femur, and a guidewire is placed at Schottle’s point [23]. However, unlike the skeletally immature patients, the guidewire is aimed proximally, from medial and distal to proximal and lateral, in the distal femur. A 40 mm femoral socket is drilled, and the guidewire is passed through the distal femur. The midportion of the graft is identified and secured to the patella with loaded suture anchors. The graft is passed between layers 2 and 3 of the knee, and the sutures at the free ends of the graft are passed through the femur following the guidewire. An interference screw is used to secure the graft in the femoral socket. The knee is brought through range of motion to check graft isometry.

### Osteochondral fracture definition and radiographic analysis

**Fig. 1:**
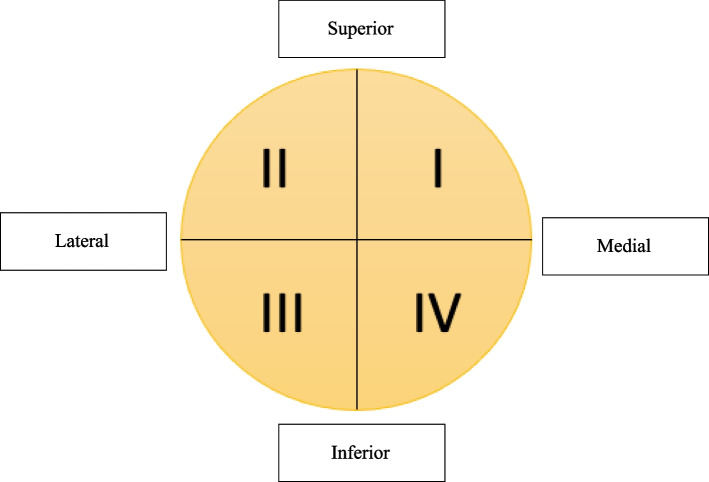
Schematic demonstrating the quadrant system used for describing the location of the patellar fractures

Patellar fractures were defined as either full-thickness chondral injuries with attached subchondral bone (osteochondral) or purely osseous injuries (without associated articular cartilage) measuring ≥ 3 mm in at least two MRI planes (sagittal, axial, or coronal). Patellar fractures without at least 3 mm of bone in two or more MRI planes were excluded from analysis. Patellar osteochondral fractures were categorized by location (Fig. 1). Patient demographic, perioperative, and post-operative clinical data were obtained via review of the electronic medical record. Traumatic injury mechanism was classified as an injury occurring after a fall or during a sporting event.

**Fig. 2:**
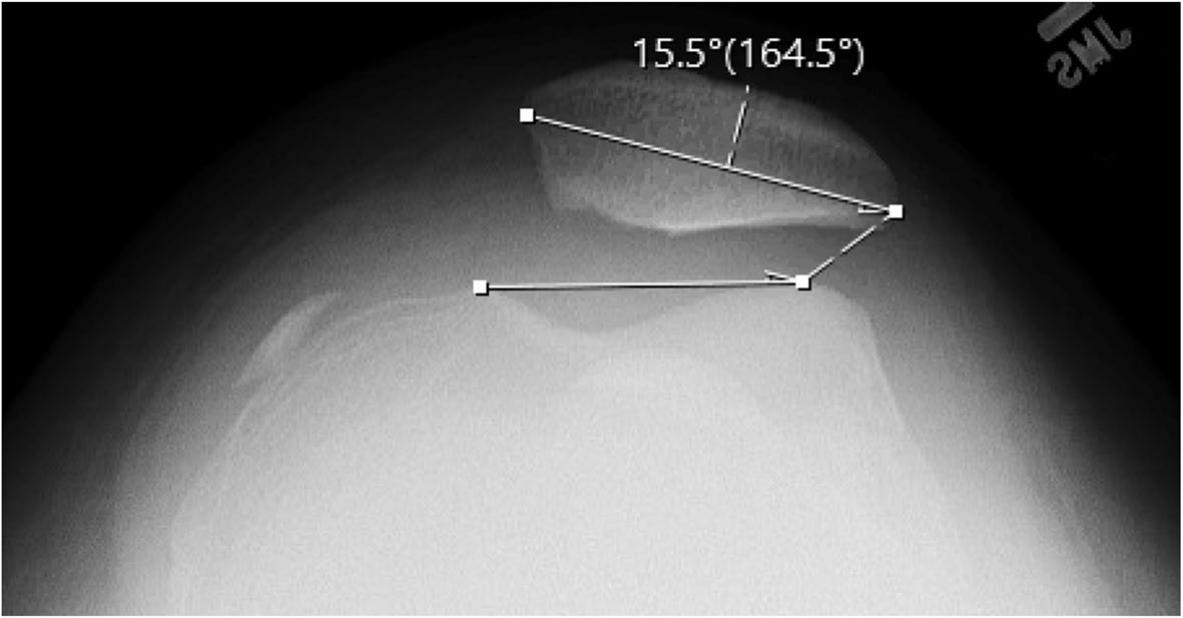
Patellar tilt as calculated on Merchant view radiograph

**Fig. 3:**
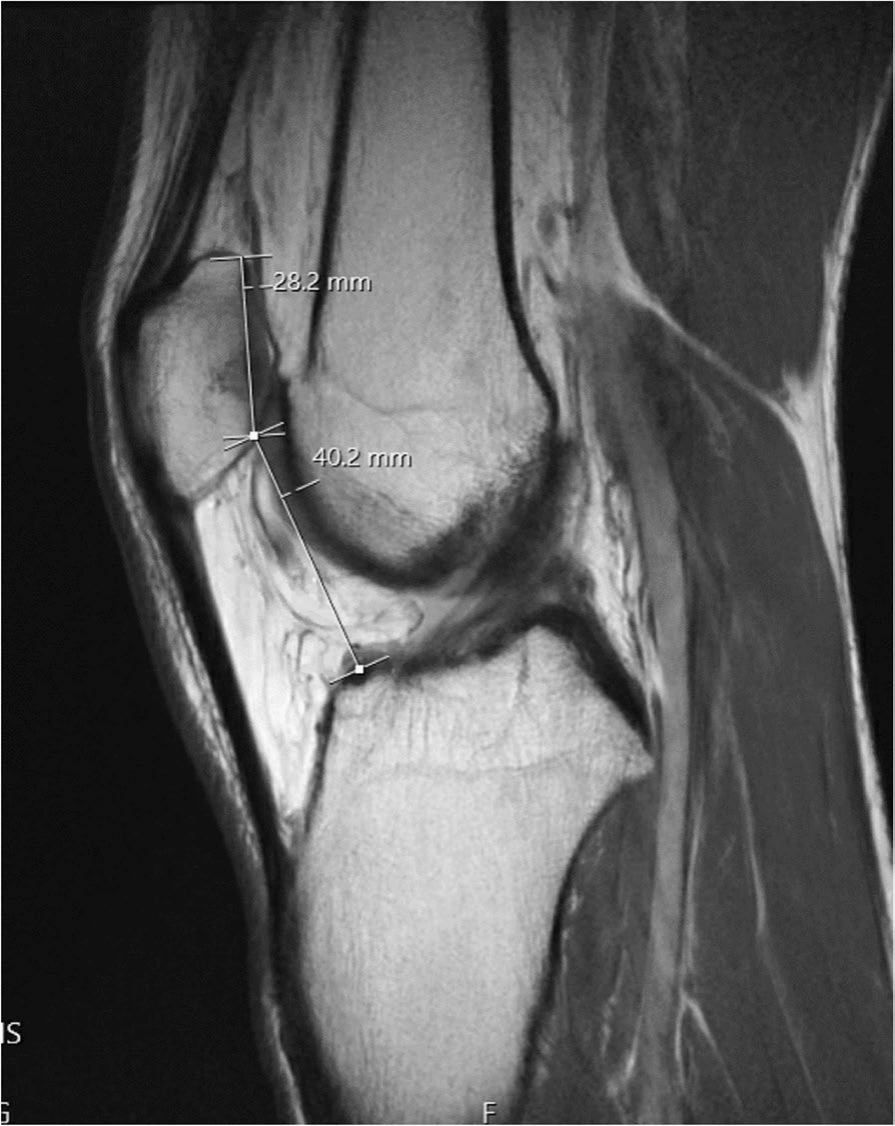
Caton Deschamps Index calculated on sagittal MRI as the ratio of the distance between the articular surface and the inferior aspect of the articular surface of the patella divided by the length of the articular surface of the patella

**Fig. 4:**
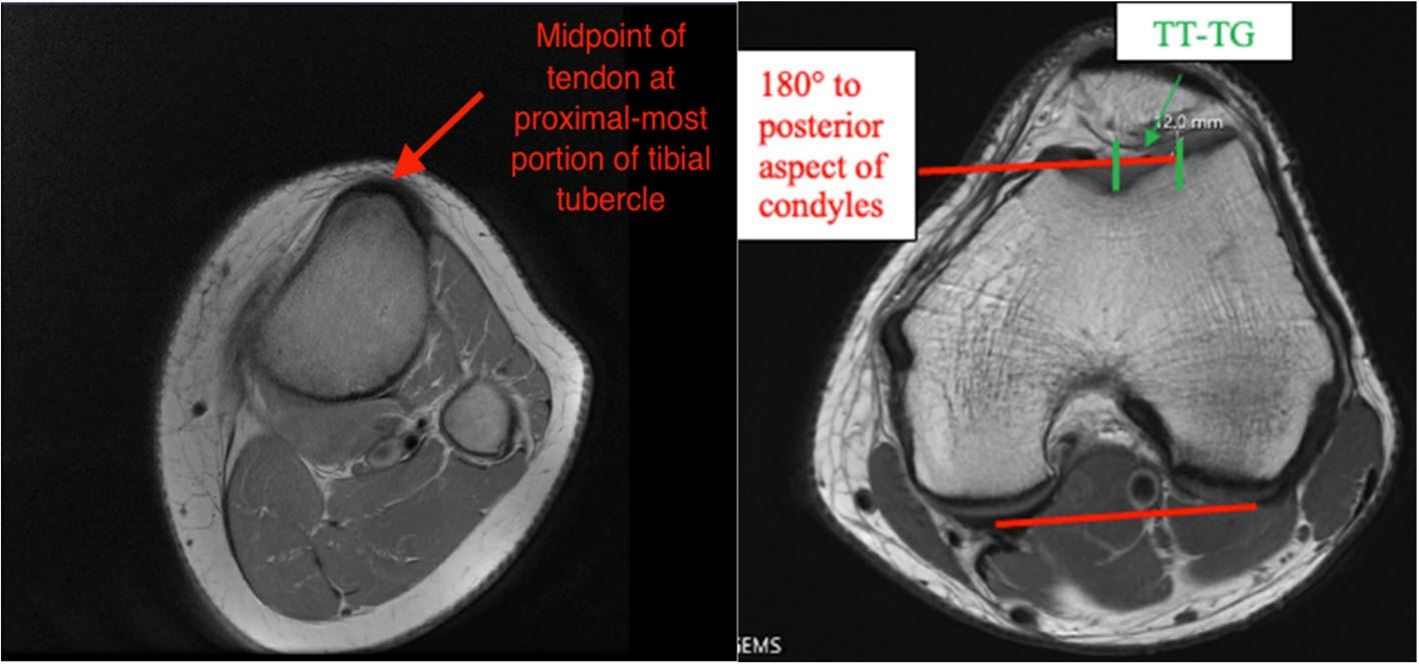
Tibial tubercle-trochlear groove distance measurement on axial MRI

Patellar tilt was calculated as the angle between a line parallel to the articular surface of the patella and a line connecting the summits of the medial and lateral femoral condyles on Merchant view radiographs (Fig. 2) [13]. The Caton Deschamps Index (CDI) was calculated as the ratio of the distance between the articular surface of the tibia and the inferior-most aspect of the articular surface of the patella divided by the length of the articular surface of the patella on sagittal MRI, one to two slices lateral to the ACL insertion (Fig. 3) [18]. The tibial tubercle-trochlear groove (TT-TG) distance was calculated as the distance between the tibial tubercle and the deepest portion of the trochlear groove on axial MRI (Fig. 4) [17]. Measurements were performed by two blinded senior orthopaedic surgery residents (.,.). Each measurement was performed twice by each observer with a break of at least 2 weeks between measurements.

### Statistical analysis

Descriptive statistics were used to evaluate patient and fracture characteristics. Means and standard deviations were used for continuous data, whereas categorical data was reported as frequencies and percentages. Data for age, TT-TG, CDI, and patellar tilt were normally distributed per the results of the Shapiro–Wilk test; therefore, unpaired t-tests were used for analysis. Data for BMI was not normally distributed based on Shapiro–Wilk test results, and Wilcoxon Rank-Sum test was used for analysis. Categorical data were analyzed with Fisher’s exact tests. Poisson regressions with robust error variance (multivariate analysis) were used to determine association between each patient characteristic and occurrence of a fracture. Two-way mixed inter- and intraclass correlation coefficients were calculated for each measurement. Data analysis was performed with STATA (Version 17.0, StataCorp). Statistical significance was determined at the α < 0.05 level.

## Results

A total of 80 patients (84 knees) were identified, 60 of whom (63 knees) remained following application of exclusion criteria. Of the patients included in the study, the mean age was 14.2 ± 1.8 years, and mean BMI was 21.9 ± 7.8. Thirty-two patients (53.3%) were female, and 30 (47.6%) were right knees (Table 1). Thirty-six patients (60%) were first-time dislocators, 49 knees (77.8%) were skeletally immature, and 45 (76.3%) had a traumatic injury mechanism. Fifty-six knees (89.9%) underwent surgery, the majority of whom underwent MPFLR (47 patients, 84%). The average follow-up was 2.7 ± 1.5 years.

**Table 1:** Demographics of all patients (*n* = 60 patients, 63 knees)

	Mean ± SD
**Age (years)**	14.2 ± 1.8
**BMI (kg/m^2)**	21.9 ± 7.8
**Average Follow-up (years)**	2.7 ± 1.5
	n (%)
**Sex**	
Male	31 (49.2)
Female	32 (50.8)
**Laterality**	
Right	30 (47.6)
Left	33 (52.4)
**Osteochondral Fracture**	
Yes	15 (23.8)
No	48 (76.2)
**First-Time Dislocators**	
Yes	36 (57.1)
No	27 (42.9)
**Skeletal Maturity**	
Mature	14 (22.2)
Immature	49 (77.8)
**Injury Mechanism**	
Traumatic	45 (76.3)
Atraumatic	14 (23.7)
**Surgery**	
Yes	56 (89.9)
No	7 (11.1)
**Type of Surgery**	
MPFLR	47 (84)
MPFLR + ORIF Patella	9 (16)

Fifteen knees (23.8%) sustained osteochondral fractures, and the majority of fractures involved the inferomedial quadrant (Zone IV, 13 fractures, 86.7%, Table 2). Thirteen of the 15 fractures (86.7%) were visible on merchant XR, whereas only half (*n* = 8/15, 53.5%) were visible on AP knee XR (Table 2). The majority (86.7%) of fractures involved articular cartilage (Table 2). There were two purely osseous fractures (no associated articular cartilage), both of which occurred in zone IV.

**Table 2:** Data for fractures

	**n (%)**
**Articular Cartilage Involvement**	
Yes	13 (86.7)
No	2 (13.3)
**Fracture Location**	
Zone 1	2 (13.3)
Zone 4	13 (86.7)
**Visible on AP Knee XR**	
Yes	8 (53.5)
No	7 (46.5)
**Visible on Merchant Knee XR**	
Yes	13 (86.7)
No	2 (13.3)
**Surgery**	
Yes	13 (86.7)
No	2 (13.3)
**Type of Surgery**	
MPFLR	8 (61.5)
MPFLR + ORIF Patella	5 (38.5)

Patients with osteochondral fractures were more likely to be male (*n* = 11/15 versus 20/48, *p* = 0.041) and skeletally immature (*n* = 15/15 versus 34/48, Table 3, *p* = 0.028). There was no difference in age between patients with osteochondral fractures (average age 13.6 ± 1.7 years) compared to those without osteochondral fractures (average age 14.4 ± 1.8 years, Table 3). There were also no differences in average BMI (21.6 ± 3.8 versus 22.1 ± 5.1, respectively, *p* = 0.82), laterality (7/15 versus 23/49 right knees, respectively, *p* = 1.0), or average follow-up (2.5 ± 1.8 years versus 2.8 ± 1.4 years, respectively, *p* = 0.25) between the two groups. There were no differences between the two groups with regard to injury mechanism (traumatic versus atraumatic, *p* = 0.15) or history of prior dislocation (Table 3, *p* = 0.23). There was also no association between sex and skeletal maturity (*p* = 0.25) or sex and age (*p* = 0.34) in the entire cohort.

**Table 3:** Demographics and measurements by fracture (*n* = 15) or no fracture (*n* = 48)

	Fracture (*n* = 15)	No Fracture (*n* = 48)	*P*-value
	Mean ± SD		
**Age (years)**	13.6 ± 1.7	14.4 ± 1.8	0.13
**BMI (kg/m^2)**	21.6 ± 3.8	22.1 ± 5.1	0.82
**Average Follow-up (years)**	2.5 ± 1.8	2.8 ± 1.4	0.25
**CDI**	1.33 ± 0.2	1.42 ± 0.2	0.079
**TT-TG**	15.7 ± 5.4	17.8 ± 4.7	0.12
**Patellar Tilt (**°**)**	12.6 ± 5.9	17.8 ± 6.8	0.021^*^
	n		
**Sex**			
Male	11	20	0.041^*^
Female	4	28	
**Laterality**			
Right	7	23	1.0
Left	8	25	
**Recurrent Dislocators**			
Yes	4	23	0.23
No	11	25	
**Skeletal Maturity**			
Mature	0	14	0.028^*^
Immature	15	34	
**Injury Mechanism**			
Traumatic	14	29	0.15
Atraumatic	1	12	

*Denotes statistical significance at *p* < 0.05

There were no differences in average CDI values (1.33 ± 0.2 versus 1.42 ± 0.2, respectively, *p* = 0.079) or TT-TG values between the two groups (15.7 ± 5.4 versus 17.8 ± 4.7, respectively, Table 3, *p* = 0.12). Patients without osteochondral fractures had increased patellar tilt (17.8 ± 6.8°) compared to those with osteochondral fractures (12.6 ± 5.9°, Table 3, *p* = 0.021).

The majority of patients with and without osteochondral fractures underwent surgical intervention (86.7% and 76.8%, respectively, *p* = 0.67). Among those with osteochondral fractures who underwent surgery (*n* = 13), 5 patients (38.5%) underwent MPFL reconstruction (MPFLR) with patellar open reduction and internal fixation (ORIF) and 8 (61.5%) underwent MPFLR only (Table 4). All of the patients who underwent MPFLR + ORIF had articular cartilage involvement (*n* = 5, 100%), whereas 62.5% (5 patients) of those who underwent MPFLR only had articular cartilage involvement (Table 4, *p* = 0.061). There were no recurrent patellar dislocations at most recent follow-up in either group. All patients with osteochondral fractures (*n* = 15) returned to sports at most recent follow up.

**Table 4:** Data for fractures treated surgically by type of surgery

	MPFLR + ORIF (*n* = 5)	MPFLR only (*n* = 8)	*P*-value
Articular Cartilage Involvement			
Yes	5 (100)	6 (75)	0.47
No	0 (0)	2 (25)	
Recurrent Dislocation			
Yes	0 (0)	0 (0)	N/A
No	5 (100)	8 (100)	

Multivariate regression analysis was also performed to control for patient factors. Statistically significant variables from univariate analysis were used as inputs to the model except for skeletal maturity, which was excluded from the model given that there were no occurrences in the reference group for this characteristic. Male sex was an independent risk factor for osteochondral fractures in our cohort (relative risk (RR) 4.8, 95% confidence interval [CI]: 1.08–20.9, *p* = 0.039), whereas patellar tilt was no longer significant following multivariate regression analysis (RR: 0.96, 95% CI: 0.90–1.01, *p* = 0.133, Table 5).

**Table 5:** Multivariate regression analysis to identify risk factors for osteochondral fracture

Input Variable	Relative Risk	*p*-value
Sex	**4.8 (M > F)**	0.039^*^
Patellar Tilt	0.96	0.13

*Denotes statistical significance at *p* < 0.05

Two-way mixed intraclass correlation coefficient values were as follows: CDI: 0.75, TTTG: 0.90, patellar tilt: 0.81, osteochondral fracture location: 0.77. Two-way mixed interclass correlation coefficient values were as follows: CDI: 0.77, TTTG: 0.76, patellar tilt: 0.90, osteochondral fracture location: 0.77.

## Discussion

We found that patellar osteochondral fractures occurred in 23% of pediatric patients with APD, and the majority of osteochondral fractures involved the inferomedial quadrant of the patella. Male sex was an independent risk factor for patellar osteochondral fractures, as males were 4.8 times more likely to sustain an osteochondral fracture than their female counterparts. In addition, skeletal immaturity was associated with osteochondral fractures in our cohort. No patients in either group had a recurrent dislocation at minimum 6-month follow-up.

Although there is a wide range of reported rates of associated osteochondral fractures in adults with APD reported in the literature (11–76%) [15, 29, 30], we found that 23% of pediatric patients with APD sustained a concomitant patellar osteochondral fracture in our cohort, similar to what has previously been described by Nietosvaara et al. in a pediatric population (29%) [14]. However, unlike Nietosvaara et al. [14], we found a higher rate of patellar osteochondral fracture after first-time dislocation (73% versus 29%).

Like Jungesblut et al. [10] and Mochizuki et al. [12], we found that the majority of osteochondral fractures of the patella involved the inferomedial quadrant. However, unlike Jungesblut et al., we did not find an association between traumatic injury mechanism and patellar osteochondral fractures, although we did find an association between male sex and skeletal immaturity with these fractures. The lack of association between traumatic injury mechanism and the presence of osteochondral fracture was a surprising finding in the present study, although patients with osteochondral fractures tended to be more likely to sustain their injuries via traumatic injury mechanism (93%) compared to patients without osteochondral fractures (71%). However, defining traumatic injury mechanism can pose a challenge, as there are inherent differences between a direct blow to the knee and a non-contact twisting injury during a sporting event. It was not possible to distinguish direct blows from non-contact twisting injuries during sports from the data available in our retrospective chart review, although future prospective studies may better evaluate the association between direct blow versus non-contact twisting injury mechanisms and the presence of patellar osteochondral fractures.

The present study builds on the existing literature by including a skeletally immature patients (average age 14.2 ± 1.8 years) who sustained patellar osteochondral fractures with articular cartilage involvement. Mochizuki et al. [12], by contrast, examined a group of older, skeletally mature patients (average age 18.5 years, 95% CI 16.1 – 20.9 years). It is important to consider skeletal maturity status when studying patellar instability in pediatric patients, as numerous prior studies have shown that skeletal immaturity is a risk factor for recurrent patellar dislocation [9, 22, 28]. Moreover, Mochizuki et al. excluded patients with isolated osteochondral fractures with articular involvement, yet articular cartilage involvement in common in patellar osteochondral fractures [21, 32].

Results of a previous histological study by Flachsmann et al. [7] may explain the higher rates of patellar osteochondral fractures in skeletally immature individuals in our cohort. Per Flachsmann et al. [7], uncalcified cartilage in skeletally immature patients weaves with subchondral bone, which renders the subchondral bone more susceptible to failure than the tidemark, which is weaker and thus more prone to failure in skeletally mature patients.

Males were 4.8 times more likely to sustain concomitant patellar osteochondral fractures than females in our cohort. This result is in line with Zheng et al. [32], who found that adolescent males were more likely to sustain a patellar chondral injury compared to adolescent females following APD. One possible explanation for this finding is that males are, on average, more skeletally immature than their female counterparts for a given chronological age [2]. In the present study, skeletal maturity was categorized as a categorical variable for the sake of statistical analysis, but in reality, skeletal maturity exists along a spectrum. Even though it is challenging to determine the degree of skeletal maturity without hand-for-bone-age XR, it is possible that the males in the present study were more skeletally immature or had a younger average skeletal age than their female counterparts, despite no differences in chronological age between these groups. Therefore, males may be more likely to sustain patellar osteochondral fractures following APD than females because they are more skeletally immature and thus have a weaker subchondral bone relative to the tidemark than females of similar chronological age.

With regard to radiographic parameters, patients without osteochondral fractures had increased patellar tilt compared to patients with osteochondral fractures. In addition, TT-TG and CDI values tended to be higher on average in the no fracture group compared to the fracture group, although this finding was not significant. Given that increased patellar tilt, CDI, and TT-TG are risk factors for patellar dislocations [1], it could be hypothesized that patients with the aforementioned risk factors could sustain a patellar dislocation via lower energy mechanisms and thus would be less likely to sustain a concomitant osteochondral fracture than those without risk factors for APD, who would likely require a greater amount of force in order to dislocate the patella.

There were two purely osseous fractures in our cohort, both of which occurred in zone IV. It is likely that these purely osseous zone IV fractures occurred as a result of a medial patellotibial ligament (MPTL) avulsion, as opposed to a medial patellofemoral ligament avulsion. The MPTL originates on the distal third of the medial border of the patella and inserts just medial to the patellar tendon insertion approximately 15 mm distal to the joint line [5]. Several prior studies have described the importance of the MPTL as a secondary restraint to lateral translation and rotation of the patella, particularly from 45° to 90° of knee flexion [16, 20], and combined MPFL and MPTL reconstruction has been shown to have good outcomes at mid-term follow up in patients with patellofemoral instability and patella alta [31].

There are several limitations to the present study. Given the retrospective nature of this study, our results are limited by selection bias, as the type and timing of surgical management were not randomized. In addition, our small sample size renders our study susceptible to type II error, particularly for variables which approached significance, such as CDI. There was also heterogeneity in our study population, as more than one third of the patients included in our study had a history of at least two patellar dislocations. However, we argue that rate of recurrent patellar dislocations in our study population is representative of pediatric patients with APD. Moreover, given the underlying assumptions of Poisson regression model, we were unable to evaluate skeletal immaturity as a risk factor for osteochondral fractures, as there were no skeletally mature patients with osteochondral fractures in our cohort. However, skeletal maturity was associated with osteochondral fractures in univariate analysis, and there was no association between skeletal maturity and gender in univariate analysis. Finally, the present study only included short-term follow-up (minimum 6 months), and future studies can include longer-term follow-up.

In conclusion, male sex is an independent risk factor for patellar osteochondral fractures, and skeletal immaturity is associated with patellar osteochondral fractures in pediatric patients. The majority of patients have good outcomes, including a low recurrence rate, at short-term follow-up.
